# Plasmodium berghei K13 Mutations Mediate *In Vivo* Artemisinin Resistance That Is Reversed by Proteasome Inhibition

**DOI:** 10.1128/mBio.02312-20

**Published:** 2020-11-10

**Authors:** Nelson V. Simwela, Barbara H. Stokes, Dana Aghabi, Matt Bogyo, David A. Fidock, Andrew P. Waters

**Affiliations:** a Institute of Infection, Immunity & Inflammation, Wellcome Centre for Integrative Parasitology, University of Glasgow, Glasgow, United Kingdom; b Department of Microbiology and Immunology, Columbia University Irving Medical Center, New York, New York, USA; c Department of Microbiology and Immunology, Stanford University School of Medicine, Stanford, California, USA; d Department of Pathology, Stanford University School of Medicine, Stanford, California, USA; e Division of Infectious Diseases, Department of Medicine, Columbia University Irving Medical Center, New York, New York, USA; NIAID/NIH

**Keywords:** malaria, *Plasmodium berghei*, *Plasmodium falciparum*, artemisinin resistance, K13, gene editing, ring-stage survival assays, parasite clearance times, proteasome, synergy

## Abstract

The recent emergence of Plasmodium falciparum parasite resistance to the first line antimalarial drug artemisinin is of particular concern. Artemisinin resistance is primarily driven by mutations in the P. falciparum K13 protein, which enhance survival of early ring-stage parasites treated with the artemisinin active metabolite dihydroartemisinin *in vitro* and associate with delayed parasite clearance *in vivo*. However, association of K13 mutations with *in vivo* artemisinin resistance has been problematic due to the absence of a tractable model. Herein, we have employed CRISPR/Cas9 genome editing to engineer selected orthologous P. falciparum K13 mutations into the *K13* gene of an artemisinin-sensitive Plasmodium berghei rodent model of malaria. Introduction of the orthologous P. falciparum K13 F446I, M476I, Y493H, and R539T mutations into P. berghei K13 yielded gene-edited parasites with reduced susceptibility to dihydroartemisinin in the standard 24-h *in vitro* assay and increased survival in an adapted *in vitro* ring-stage survival assay. Mutant P. berghei K13 parasites also displayed delayed clearance *in vivo* upon treatment with artesunate and achieved faster recrudescence upon treatment with artemisinin. Orthologous C580Y and I543T mutations could not be introduced into P. berghei, while the equivalents of the M476I and R539T mutations resulted in significant growth defects. Furthermore, a *Plasmodium*-selective proteasome inhibitor strongly synergized dihydroartemisinin action in these P. berghei K13 mutant lines, providing further evidence that the proteasome can be targeted to overcome artemisinin resistance. Taken together, our findings provide clear experimental evidence for the involvement of K13 polymorphisms in mediating susceptibility to artemisinins *in vitro* and, most importantly, under *in vivo* conditions.

## Abstract (precis)

Recent successes in malaria control have been seriously threatened by the emergence of Plasmodium falciparum parasite resistance to the frontline artemisinin drugs in Southeast Asia. P. falciparum artemisinin resistance is associated with mutations in the parasite K13 protein, which associates with a delay in the time required to clear the parasites upon drug treatment. Gene editing technologies have been used to validate the role of several candidate K13 mutations in mediating P. falciparum artemisinin resistance *in vitro* under laboratory conditions. Nonetheless, the causal role of these mutations under *in vivo* conditions has been a matter of debate. Here, we have used CRISPR/Cas9 gene editing to introduce K13 mutations associated with artemisinin resistance into the related rodent-infecting parasite, Plasmodium berghei. Phenotyping of these P. berghei K13 mutant parasites provides evidence of their role in mediating artemisinin resistance *in vivo*, which supports *in vitro* artemisinin resistance observations. However, we were unable to introduce some of the P. falciparum K13 mutations (C580Y and I543T) into the corresponding amino acid residues, while other introduced mutations (M476I and R539T equivalents) carried pronounced fitness costs. Our study provides evidence of a clear causal role of K13 mutations in modulating susceptibility to artemisinins *in vitro* and *in vivo* using the well-characterized P. berghei model. We also show that inhibition of the P. berghei proteasome offsets parasite resistance to artemisinins in these mutant lines.

## INTRODUCTION

Artemisinin (ART)-based combination therapies (ACTs) have been at the forefront of globally coordinated efforts to drive down the burden of malaria. A pharmacodynamic hallmark of ARTs and their derivatives is that they are highly active and fast acting against blood stages of malaria parasites. These drugs can achieve up to 10,000-fold parasite reductions in the first replication cycle upon drug exposure (1). Such is the effectiveness of ARTs that recently reported reductions in malaria morbidity and mortality are, indeed, partly attributed to ACTs (2). The use of ARTs in combination therapies originated from early clinical trials, which showed that despite achieving faster parasite clearance, ART monotherapies resulted in recrudescence rates of up to 40% (3). ACTs deliver a pharmacological cure by taking advantage of ARTs to rapidly clear the parasite biomass in the early days of treatment while relying on the partner drug to eliminate residual parasites (4). So far, ACTs remain highly effective in Sub-Saharan Africa, the region that harbors the highest disease burden, with efficacy rates of >98% (2). Nevertheless, ACTs have been threatened by the emergence of Plasmodium falciparum resistance to ARTs in Southeast Asia, and resistance has the potential to spread to other regions of malaria endemicity, as has been a historical trend with earlier first-line antimalarial drugs (2, 5–7). Recently, locally derived K13 variants that are able to mediate ART resistance *in vitro* have been identified in P. falciparum parasites in French Guiana and in Rwanda (8, 9), further illustrating the emergent threat to ART efficacy. Moreover, an aggressive expansion of a parasite lineage carrying the genetic determinants of resistance to both ART derivatives and the ACT partner drug piperaquine has been reported across Southeast Asia, resulting in a dramatic loss of clinical efficacy (10–13).

Clinically, P. falciparum resistance to ARTs manifests as reduced *in vivo* parasite clearance upon treatment with ACTs or ART monotherapies (2, 14, 15). These clearance rates are based on the Worldwide Antimalarial Resistance Network (WWARN) parasite clearance estimator (16), which quantifies relative resistance by estimating parasitemia lag phases and clearance half-lives upon treatment with artesunate (AS) or ACTs. This involves *in vivo* quantification of viable parasitemia (in patients) upon treatment with AS (2 to 4 mg/kg body weight/day) or ACTs at specified time intervals and subsequent calculation of parasite densities as a function of time (16). The parasite clearance estimator has been used to generate substantial baseline data that classify ART resistance as parasite clearance half-lives of >5.5 h and ART sensitivity as parasite clearance half-lives of <3 h (17, 18). However, interpretation of clearance half-lives can be confounded by differences in initial parasite biomass, the efficacy of the partner drug, and the level of host immunity (17, 19). Moreover, this *in vivo* phenotype does not correlate with decreased susceptibility to dihydroartemisinin (DHA) in standard growth inhibition assays where P. falciparum parasites (which have a ∼48-h intraerythrocytic developmental cycle) are exposed to the drug for a total of 72 h (15, 20, 21). The ring-stage survival assay (RSA), where highly synchronized early-ring-stage parasites (0 to 3 h postinvasion) are exposed for a short period of time (3 to 6 h) to DHA (at the pharmacologically relevant concentration of 700 nM), provides an improved correlate for the *in vivo* delayed parasite clearance phenotype and has been the principal *in vitro* assay for determining P. falciparum resistance to ARTs (22, 23). At the genetic level, polymorphisms in the P. falciparum K13 propeller domain have been strongly associated with ACT treatment failure (21, 24) and also correlate with delayed parasite clearance *in vivo* and increased parasite survival *in vitro* in RSAs (25–27). Reverse genetic approaches have been successfully used to show that the P. falciparum K13 mutations M476I, R539T, I543T, Y493H, and C580Y can confer DHA resistance *in vitro*, as defined by >1% survival in RSAs (28, 29). However, the parasite genetic background as well as underlying polymorphisms in drug resistance determinants such as *pfcrt* (*P. falciparum* chloroquine resistance transporter) and *pfmdr2* (*P. falciparum* multidrug resistance protein-2) may play a role either by modulating different levels of susceptibility to DHA or by providing a suitable biological landscape upon which these K13 mutations are more likely to arise (25, 28).

ART resistance as typified by the “delayed clearance phenotype” is, however, still classified as “partial resistance,” primarily because most patients with parasites harboring the phenotype effectively clear the infection when an effective partner drug is used or duration of monotherapy is extended (4). ART partial resistance is, therefore, confirmed or suspected when patients carry parasites with certain K13 mutations, display a parasite clearance half-life of >5.5 h, or are microscopically smear positive on day three after initiation of treatment (2, 4). The full extent to which these parameters predict subsequent ACT treatment failure or define ART resistance remains an area of continuing debate (30–35). The definition of ART resistance in these contexts would thus benefit from experimentally accessible *in vivo* models that would help interrogate ART parasite susceptibility parameters, including clearance half-lives, recrudescence rates, and treatment failures. Such models would allow for a genetic dissection of the role of K13 mutations in mediating resistance *in vivo* in the absence of confounding factors such as secondary genetic factors and/or host factors (25, 28). Currently, the K13 C580Y polymorphism is the most prevalent and dominant ART-resistant mutation in Southeast Asia (14, 36). A recent genetic cross of the K13 C580Y ART-resistant line with an *Aotus* monkey-infecting P. falciparum strain provided evidence, in this nonhuman primate model, that parasites carrying the C580Y mutation can display increased survival in *in vitro* RSAs with no accompanying *in vivo* ART resistance (37).

Moreover, P. falciparum drug resistance mutations are known to often associate with significant fitness costs that limit the prevalence and eventual propagation of resistance-conferring alleles in natural infections. For example, mutations in the P. falciparum chloroquine (CQ) resistance transporter (*pfcrt*) that modulate resistance to CQ massively expanded when CQ was in use in the 1970s but eventually were outcompeted and replaced with parasites carrying wild-type alleles in African high-transmission settings following withdrawal of CQ use (38, 39). Similarly, P. falciparum K13 mutations have been shown to carry *in vitro* fitness costs; however, the degree to which a given mutation is detrimental for growth seems to depend on the parasite genetic background (40). Relative to other K13 mutations, P. falciparum R539T and I543T mutant parasites that are associated with the highest RSA survival rates (23, 28) and most significant delays in parasite clearance (41) also carried the most pronounced fitness costs (40). Intriguingly, the most prevalent K13 mutation in Southeast Asia, C580Y, was fitness neutral *in vitro* when gene edited into recent Cambodian clinical isolates, whereas it displayed a significant growth defect when introduced into ART-susceptible parasites isolated before ARTs were widely deployed (40, 42). Recently, it was demonstrated that P. falciparum K13 localizes to the parasite cytostomes and other intracellular vesicles and plays a role in parasite hemoglobin endocytosis and trafficking to the lysosome-like digestive vacuole (43–45). K13 mutations are thought to lead to a partial loss of protein function, which subsequently impairs hemoglobin endocytic uptake, thereby lessening ART activation and conferring ART resistance (43). This has pointed toward a K13-mediated hemoglobin-centric mechanism of ART resistance, which could possibly be shared with other drugs such as CQ that act by binding to heme moieties in the digestive vacuole, following cytostome-mediated hemoglobin endocytosis (44, 46–48). Of note, mutant K13-mediated ART resistance phenotypes are associated with upregulated cellular stress responses, which can be targeted by selective inhibition of the parasite 26S proteasome (49, 50).

Here, we report the *in vitro* and *in vivo* phenotypes of orthologous P. falciparum K13 mutations that were gene edited into an *in vivo* rodent model of malaria, Plasmodium berghei. We profiled the fitness of these P. berghei K13 mutant parasites relative to their isogenic wild-type counterparts as well as their sensitivity to combinations of DHA and proteasome inhibitors. Our data provide evidence that K13 mutations are causal for reduced susceptibility to ARTs in an *in vivo* model and link these mutations to *in vitro* and *ex vivo* phenotypes. Our findings also demonstrate that inhibition of the *Plasmodium* proteasome is an effective strategy to restore ART action in resistant parasites that survive treatment with ART alone.

## RESULTS

### CRISPR/Cas9-mediated introduction of P. berghei orthologous K13 mutations and *in vivo* mutant enrichment by AS.

**FIG 1:**
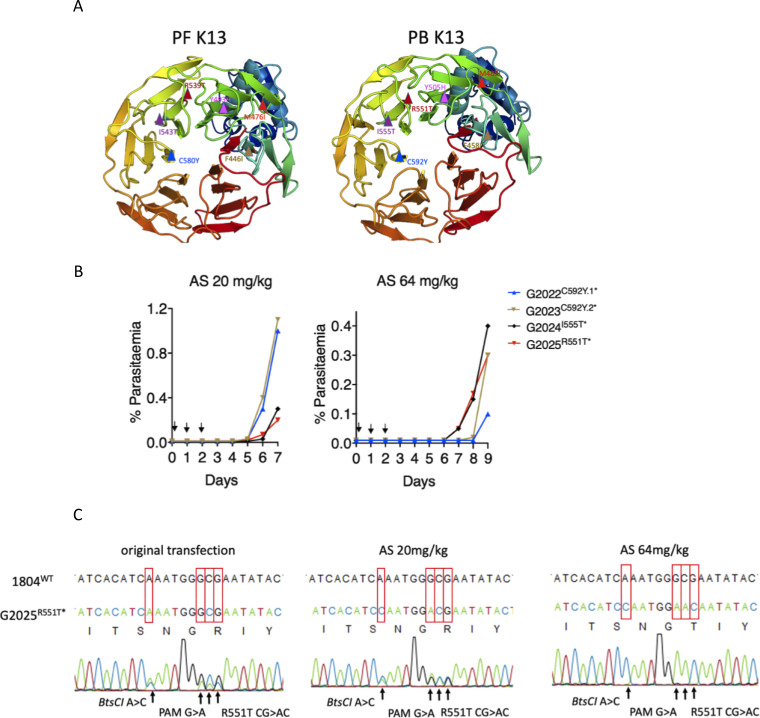
Introduction of orthologous K13 nucleotide substitutions in P. berghei. (A) Three-dimensional homology model of P. falciparum (PF3D7_1343700) and P. berghei (PBANKA_1356700) K13 for amino acid residues 350 to 726 and 362 to 738, respectively. P. falciparum K13 mutation sites (F446I, M476I, Y493H, R539T, I543T, and C592Y) are indicated in the structure on the left, and P. berghei orthologous mutation sites are modeled on the right. Models were created in SWISS-MODEL using PDB template 4zgc.1.A. Structures were visualized and annotated using PyMOL 2.3. (B) Parasitemia growth curves monitoring recrudescence of the G2022, G2023, G2024, and G2025 lines upon artesunate (AS) challenge. Mice were infected with 2 × 10^7^ parasites by i.p. injection on day 0. Treatment with AS was commenced ∼3 h postinfection by i.p. injection and was continued for three consecutive days as indicated by arrows. Parasitemia was monitored microscopically until recrudescence was observed. Mice were bled when the parasitemia was less than 1.5% to minimize competition from wild-type parasites in case mutants carried growth defects. (C) Sanger sequencing of bulk DNA from the G2025 R551T line showing selective enrichment of this mutation upon AS treatment at 20 or 64 mg/kg. Enrichment of this mutation was also observed in the RFLP analysis (see also Fig. S2B in the supplemental material).

To generate P. berghei mutant parasites carrying orthologous P. falciparum K13 mutations, we attempted to introduce P. berghei equivalents of five P. falciparum K13 mutations (M476I, Y493H, R539T, I543T, and C580Y) that by reverse genetics were previously shown to confer enhanced P. falciparum survival in *in vitro* RSAs (28). We also introduced the equivalent of the F446I mutation that is predominant in Southern China along the Myanmar border (14). These mutations are all validated determinants of reduced P. falciparum susceptibility to ARTs (4). Structural homology modeling revealed that P. berghei and P. falciparum K13 (PBANKA_1356700 and PF3D7_1343700, respectively) are highly conserved (∼84% sequence identity overall) at the C-terminal propeller domain, especially where resistance-conferring mutations localize (Fig. 1A). P. berghei K13 carries 12 extra amino acids, resulting in 738 amino acids for P. berghei compared to 726 for P. falciparum. However, modeling suggests that the extra amino acids in P. berghei do not change the overall propeller structure of K13 or the amino acid identity at the orthologous positions of the mutations examined in this study (Fig. 1A; see also Fig. S1A and B in the supplemental material). Using a CRISPR/Cas9 system (Fig. S2A) (46), we designed Cas9 plasmids carrying single guide RNAs (sgRNAs) to target the P. berghei K13 locus with corresponding homology repair templates. The repair templates carried the mutations of interest as well as silent mutations that inactivated the protospacer adjacent motif (PAM) and introduced restriction sites for restriction fragment length polymorphism (RFLP) analyses (see Table S1). Electroporation of the plasmids pG1004 (C592Y), pG1005 (I555T), and pG1006 (R551T) into the K13 wild-type P. berghei 1804cl1 line yielded edited parasites (G2022^C592Y.1^*, G2023^C592Y.2^*, G2024^I555T^* and G2025^R551T^*) with calculated 13.4%, 18.5%, 7.7%, and 30.0% efficiencies, respectively, by RFLP analysis (see Fig. S2B; Table S1). Intriguingly, bulk DNA sequencing of these transformed parasites revealed that only the G2025^R551T^* line carried sequence traces for the R551T amino acid substitution and accompanying silent mutations (Fig. S3A), while the rest had traces only of the silent mutations (Fig. S3B and C). Our prior studies with refractory mutations have also revealed the parasite’s ability to restrict CRISPR/Cas9-mediated double-stranded break repair to the region immediately proximal to the cut site, thereby capturing the silent mutations without extending to nearby deleterious single nucleotide polymorphisms (SNPs) (46). We suspect this is a consequence of very short resection events (51). These data suggested that the C592Y and I555T mutations either result in extremely slow growing parasites or are entirely lethal in P. berghei. We attempted to clone the G2025^R551T^* line by limiting dilution, but this could not be achieved, possibly due to the low mutant population (30.0%) combined with a potentially low growth rate of the mutants compared to that of wild-type parasites.

10.1128/mBio.02312-20.1 FIG S1 Schematic and amino acid alignment of P. falciparum and P. berghei K13. (A) P. falciparum K13 protein showing amino acid positions and the protein domains. Positions of K13 mutations that have been investigated in this study are indicated. Equivalent amino acid positions for P. berghei are indicated in parallel at the bottom (in blue). (B) Protein alignment of P. falciparum and P. berghei K13 showing conservation at the mutation sites. Alignments were carried out using Clustal Omega protein alignment tool. Conserved residues are indicated by “*.” Download FIG S1, TIF file, 2.9 MB. Copyright © 2020 Simwela et al. 2020 Simwela et al. This content is distributed under the terms of the Creative Commons Attribution 4.0 International license.

10.1128/mBio.02312-20.2 FIG S2 CRISPR/Cas9 editing strategy and RFLP analysis of P. berghei K13 mutant lines. (A) Schematic of CRISPR/Cas9 strategy used to introduce K13 mutations into P. berghei asexual blood-stage parasites. For this, 20-bp sgRNA targeting regions within 0 to 30 bp of the mutation site (see Tables S1 and S2) were designed to contain *Esp3I* digestion overhangs and were cloned into the Cas9 plasmid ABR099, as previously described (46). Donor templates were generated by overlapping extension PCR as described in Materials and Methods and were subsequently cloned into the ABR099 plasmids carrying the appropriate sgRNA (Tables S1 and S2) at the *HincII* linker site. The donor templates carried the mutation of interest as well as silent mutations to introduce a restriction site for RFLP analysis and to inactivate the PAM site recognition sequence (as illustrated in the schematic). Details of plasmids, sgRNA, and lines generated are in Table S1. (B) RFLP analysis of transfected parasite populations before and after challenge with artesunate (AS) at 20 or 64 mg/kg for the G2022 (C592Y.1), G2023 (C592Y.2), G2024 (I555T), and G2025 (R551T) lines. RFLP analysis was carried out using the indicated restriction enzymes on PCR fragments amplified from genomic DNA of transfected parasites. PCRs used primers that bound on either side of the donor DNA (Table S1 and S2). (C) RFLP analysis of parasite bulk populations for the G1957 (F458I), G1979 (Y505H), and G1989 (M488I) transfected lines. RFLP analysis was carried out using the indicated restriction enzymes. In both RFLP analyses, “*” indicates that these lines were uncloned. Download FIG S2, TIF file, 2.8 MB. Copyright © 2020 Simwela et al. 2020 Simwela et al. This content is distributed under the terms of the Creative Commons Attribution 4.0 International license.

10.1128/mBio.02312-20.3 FIG S3 DNA sequence and further RFLP analysis of P. berghei K13 mutant lines. (A) Sequencing analysis of the G2025 line showing the presence of traces for both the silent mutations and the R551T substation in the original transfection. DNA sequencing showing the absence of the C592Y and I555T nucleotide substitutions and the presence of minor traces of the silent mutations in the G2023 (B) and G2024 (C) transfected lines; “*” on the transfectant parasite lines indicates that the line was not cloned. (D) RFLP analysis of the G2042 (C592Y, sgRNA 2, TAT codon), G2043 (C592Y, sgRNA 2, TAC codon), and G2044 (C592Y, sgRNA 1, TAC codon) transfected lines (Table S1), showing further unsuccessful attempts to introduce the C592Y in P. berghei. RFLP analysis of the G2045 control line (C592C, sgRNA 1, silent mutations control) where editing was readily achieved is shown for comparison. (E) Sequencing analysis of the G2045 line showing successful editing with high efficiency to introduce silent mutations without the C592Y substitution. DNA sequence analysis showing high efficiency editing to introduce silent mutations and mutations of interest in the G1957 (F458I) (F), G1989 (M488I) (G), and G1979 (Y505H) (H) lines. (I) RFLP analysis with indicated restriction enzymes for the cloned parasite lines G1957 (F458I), G1979 (Y505H), G1989 (M488I), and G2025 (R551T, AS 64 mg/kg). Download FIG S3, TIF file, 2.8 MB. Copyright © 2020 Simwela et al. 2020 Simwela et al. This content is distributed under the terms of the Creative Commons Attribution 4.0 International license.

10.1128/mBio.02312-20.7 TABLE S1 Plasmids, generated lines, transfection efficiencies, and outcome genotypes. RFLP analysis of the bulk transfected parasites was carried out on PCR fragments amplified using diagnostic PCR primers exterior of the donor template: GU5300 plus GU5301 (1,111 bp) for K13 and GU5186 plus GU4895 (946 bp) for UBP-1. Download Table S1, XLSX file, 0.1 MB. Copyright © 2020 Simwela et al. 2020 Simwela et al. This content is distributed under the terms of the Creative Commons Attribution 4.0 International license.

In earlier efforts to introduce UBP-1 mutations in P. berghei, we found that preemptive drug pressure to which the engineered mutation is anticipated to confer a protective advantage can selectively enrich for the mutant in a mixed, transfected parasite population, even when the mutant population is <1% in the mixture (46). Using this approach, we subjected a larger inoculum (2 × 10^7^) of the G2022^C592Y.1^*, G2023^C592Y.2^*, G2024^I555T^*, and G2025^R551T^* lines to AS at 20 or 64 mg/kg to see if any enrichment in the recrudescent parasite populations could be achieved (Fig. 1B). Indeed, AS at both 20 and 64 mg/kg specifically enriched the R551T mutant population in the G2025^R551T^* line from 30.0% in the initial transfection to 49.7% at AS 20 mg/kg and >99% at 64 mg/kg (Fig. 1C; Fig. S2B and Table S1). In contrast, apart from a minor enrichment that was observed for the G2024^I555T^* line, no useful enrichments in both the G2022^C592Y.1^* and G2023^C592Y.2^* lines were observed by RFLP at either concentration of AS (Fig. S2B; Table S1). Furthermore, no I555T or C592Y amino acid substitution traces could be seen after population-level DNA sequencing of these lines. These data further supported the relative nonviability of P. berghei parasites bearing K13 C592Y and I555T mutations. In agreement with the above-described observations, further attempts to introduce the C592Y mutation using a different sgRNA and/or different codons for the tyrosine residue in the donor template (TAT or TAC) were also unsuccessful. We did, however, observe >90% editing efficiency when introducing only silent mutations that maintained the C592C wild-type genotype in the donor template (Fig. S3 and E; Table S1). This, plus other unsuccessful attempts to generate the I555T mutant, further implies that these two K13 mutations are not viable in P. berghei. Meanwhile, transfection of the P. berghei 1804cl1 line with pG983 (F458I), pG984 (Y505H), and pG1008 (M488I) successfully introduced these mutations in P. berghei K13, yielding the G1957^F458I^*, G1979^Y505H^*, and G1989^M488I^* lines with >93% efficiencies, as confirmed by RFLP analysis (Fig. S2C; Table S1) as well as population-level DNA sequencing (Fig. S3F, G, and H). These three lines (G1957^F458I^*, G1979^Y505H^*, and G1989^M488I^*) and the G2025^R551T^* AS 64 mg/kg-challenged line were all cloned by limiting dilution. Mutations were further confirmed by RFLP analysis (Fig. S3I) and sequencing. The V2721F UBP-1 mutant line, which we previously found to mediate reduced susceptibility to ARTs in P. berghei (46), was also generated in the 1804cl1 background and cloned (Table S1).

### P. berghei K13 mutants display reduced susceptibility to DHA in 24-h assays and increased survival in P. berghei-adapted RSAs.

**FIG 2:**
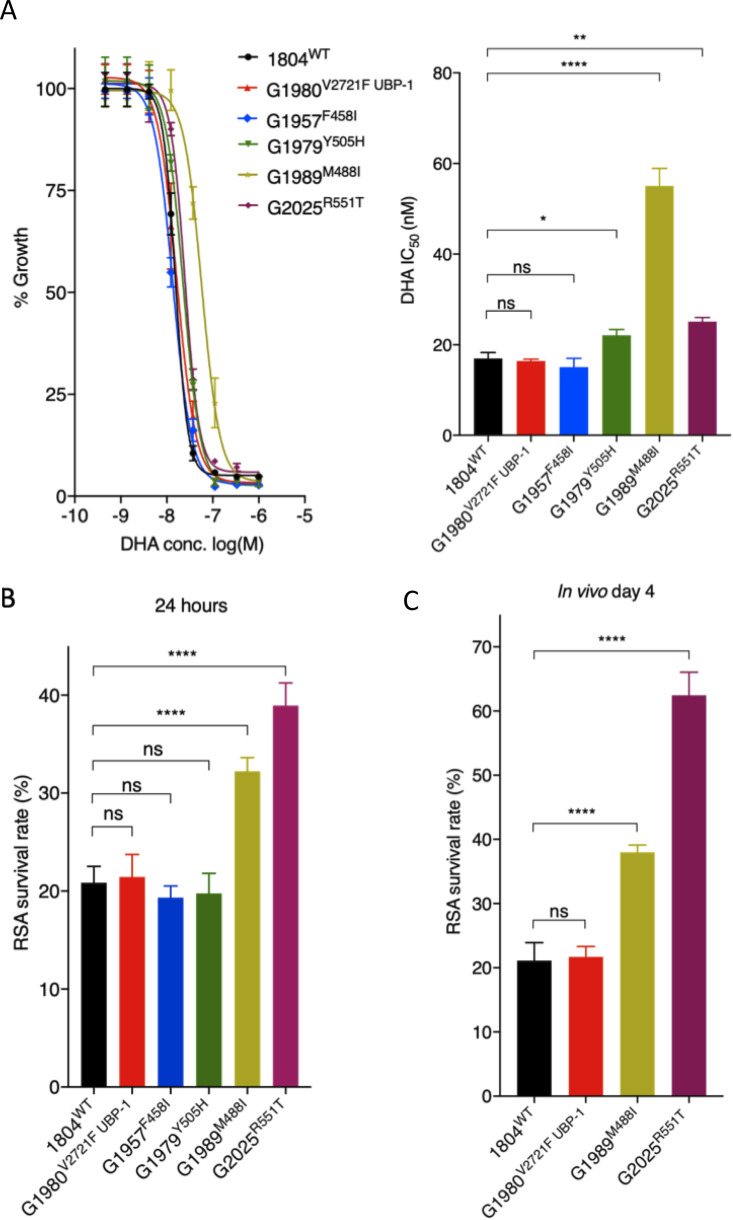
*In vitro* and *ex vivo* susceptibility of P. berghei K13 mutants to DHA. (A) DHA dose-response curves and IC_50_ values for P. berghei K13 mutant lines compared to those of the wild-type 1804^WT^ and the UBP-1 G1980^V2721F^ mutant lines. (B) Survival of P. berghei K13 mutant lines in the P. berghei RSA. Results show the percentages of synchronized early ring-stage parasites (1.5-h postinvasion) that survived a 3 h exposure to 700 nM DHA relative to DMSO-treated parasites. Survival was quantified 24 h posttreatment by flow cytometry analysis based on Hoechst 33258 DNA staining and mCherry expression. (C) *In vivo* RSA survival for two K13 mutant lines (G1989^M488I^ and G2025^R551T^) compared to that of the wild-type (1804^WT^) and UBP-1 mutant (G1980^V2721F^) controls. After *in vitro* exposure to DHA or DMSO as described above, parasites were i.v. injected back into mice as described in Materials and Methods. Parasitemia was quantified by flow cytometry analysis of mCherry expression on day 4 after i.v. injection, from which percentage survival rates were calculated. Error bars show standard deviations calculated from three biological repeats. Statistical significance (compared to the 1804^WT^ line) was calculated using one-way analysis of variance (ANOVA) alongside the Dunnett’s multiple-comparison test. ns, not significant; *, *P* < 0.05; **, *P* < 0.01; ****, *P* < 0.0001.

Unlike that for P. falciparum, P. berghei can only be maintained in one blood-stage cycle *in vitro*, which restricts drug susceptibility assays to one 24 h developmental cycle. Drug susceptibility readouts are therefore based on single-generation flow cytometry quantification of schizont maturation (46, 52, 53). Using this approach, we aimed to characterize the DHA dose-response profiles of the P. berghei K13 mutants compared to those of wild-type parasites or to a previously reported UBP-1 mutant with reduced ART susceptibility (46). Interestingly, in contrast to the equivalent P. falciparum K13 mutants, P. berghei M488I, R551T, and Y505H K13 mutant parasites displayed reduced susceptibility to DHA in standard growth inhibition assays with 3.3-, 1.4-, and 1.2-fold 50% inhibitory concentration (IC_50_) increases, respectively, compared to that of isogenic K13 wild-type parasites (Fig. 2A). The P. berghei F458I K13 mutant displayed equal sensitivity to DHA as the wild-type and the UBP-1 V2721F mutant (Fig. 2A), in agreement with our previous observations (46). These data suggest that, despite being limited to a single-cycle 24 h exposure, the P. berghei standard assay can distinguish even modestly ART-resistant parasites from sensitive ones. We next investigated the DHA susceptibility of early ring-stage P. berghei K13 mutant parasites by adapting the P. falciparum RSA (22). The P. falciparum RSA relies on exposure of early ring-stage parasites (0 to 3 h postinvasion) to 700 nM DHA for 4 to 6 h, followed by assessment of viability in the 2nd life cycle. This protocol allows drug-exposed parasites to reinvade fresh red blood cells. With this approach, current RSA parameters define *in vitro* ART resistance as survival of ≥1% and ART sensitivity as <1% survival (22). Using a similar approach, we exposed ∼1.5-h postinvasion K13 mutant P. berghei ring-stage parasites to DHA at 700 nM for 3 h (to accommodate for the shorter life cycle in P. berghei). Viability was assessed 24 h later by flow cytometry-based quantification of schizont maturation and mCherry expression. Interestingly, we observed that a significant fraction of P. berghei wild-type parasites survived exposure to DHA at 700 nM, with percentage survival rates of ∼20.9% (Fig. 2B). This is in agreement with our previous observations that P. berghei is less susceptible to ARTs than P. falciparum (46, 54). Both the UBP-1 mutant and F458I or Y505H K13 mutant parasites had the same survival rates as the wild-type line, whereas the M488I and R551T mutants exhibited significantly higher survival rates (32.3% or 39.0%, respectively, *P* < 0.001) (Fig. 2B). This is consistent with previous reports that, in P. falciparum, the R539T and I543T mutations are associated with the highest rates of RSA survival (28). However, we noted inconsistencies between drug susceptibility data of the mutants in the two *in vitro* tests (standard 24-h assay and adapted P. berghei RSA). This might result from the inability to maintain P. berghei in long-term culture and extend the analysis. We therefore developed a modified *in vivo* RSA, where we injected wild-type, UBP-1 V2721F, M488I, and R551T parasites back into mice 24 h after dimethyl sulfoxide (DMSO) or DHA exposure in the RSA as described above and then assessed viability by quantifying *in vivo* parasitemia on day 4. Remarkably, percentage survival in the R551T mutant parasites significantly increased from ∼39.0% (24 h readout) to ∼62.5%, while M488I mutant parasite survival increased from ∼32.3% (24 h readout) to ∼38.0% (Fig. 2C). In contrast, the percentage survival of the wild-type and UBP-1 mutant lines did not significantly change in the extended assay, despite the minor growth defect in the UBP-1 mutant, demonstrating that the P. berghei *in vitro* RSA and standard growth inhibition assays with 24-h readouts may be less robust in quantifying resistance phenotypes, especially if mutant parasites are less fit (Fig. 2C).

### P. berghei K13 mutants mimic the delayed parasite clearance phenotype *in vivo* upon AS treatment and achieve faster recrudescence than wild-type parasites at high ART doses.

**FIG 3:**
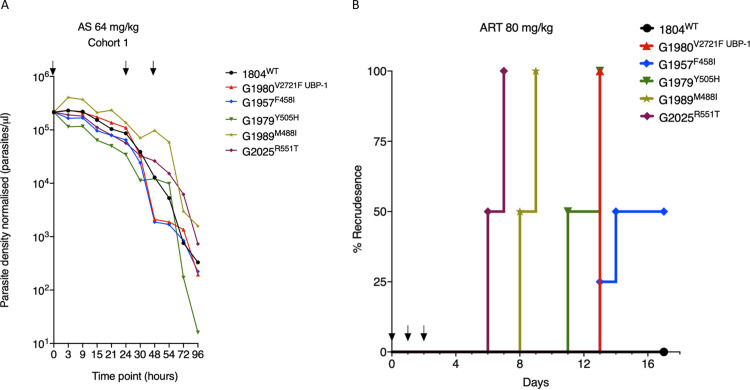
*In vivo* clearance and recrudescence rate of P. berghei K13 mutants following treatment with AS or ART. (A) Parasite clearance curves in mice infected with P. berghei K13 mutant lines following treatment with AS. Six mice (in each of four cohorts) were infected with 10^5^ parasites of each of the four K13 mutants, the UBP-1 mutant, and wild-type control on day 0. On day 5, at a parasitemia of ∼10%, mice were dosed with AS at 64 mg/kg body weight. Day 5 was the designated 0 h time point for the dosing regimen. Parasite density per microliter of blood was quantified based on absolute counts of mCherry-positive parasites at staggered time points for each of the two cohorts, with 5 time points in the first 24 h (corresponding to at least 3 h interval coverage between the two cohorts) and at least once daily thereafter. Mice were dosed three times at 0, 24, and 48 h as indicated by arrows. Concurrent thin blood smears were prepared at each time point for microscopic analysis (Fig. S4). (B) Kaplan-Meier plots of recrudescence in wild-type and UBP-1 mutant controls compared to that of K13 mutants. A modified Peters’ 4-day suppressive test was used to monitor susceptibility of the K13 mutants to 80 mg/kg ART, a dose that effectively suppresses wild-type parasites for up to 18 days. Groups of three (UBP-1 mutant, 1804^WT^) or four mice (K13 mutants) were infected with 1 × 10^6^ parasites on day 0. ART treatment was initiated ∼3 h later and continued every 24 h for three consecutive days (treatment days shown by arrows). Parasitemias were monitored by microscopic analysis of Giemsa-stained blood smears up to day 18 (Table S3). Recrudescence rates were plotted as the proportion of mice in the treatment groups that became smear positive on every individual day for the 18 days of follow-up.

We next investigated the *in vivo* parasite clearance rates of P. berghei K13 mutant parasites in infected mice treated with AS. Mice were infected with a fixed inoculum of K13 and UBP-1 mutant parasites (10^5^) in four cohorts, and parasitemias were allowed to rise to ∼10%. This was followed by dosing with AS at 64 mg/kg body weight, which is slightly higher than the equivalent of the maximal human clinical dose of 4 mg/kg (mouse equivalent = 49.2 mg/kg) to accommodate for the reduced ART susceptibility observed in P. berghei parasites. Parasitemias were quantified by flow cytometry (based on mCherry positivity) and microscopic analysis every 3 h for the first 24 h and at least once after the second and third doses at 24 and 48 h, respectively. Plotting parasite density in P. berghei K13 and UBP-1 mutant parasites against time revealed that in the first 24 h of sampling, parasite clearance kinetics did not sufficiently discriminate K13 or UBP-1 mutant parasites from the wild type. However, as the majority of dying parasites were being cleared by the host and mice received further doses, extended analysis revealed that P. berghei M488I and R551T mutant parasites consistently and significantly persisted compared to wild-type, F458I, Y505H, and UBP-1 mutant parasites (Fig. 3A; see also Fig. S4). Starting AS treatment at a high initial parasitemia (∼10%) also ensured that a good proportion of parasites would be within the early ring-stage window and, therefore, would be expected to preferentially survive the first AS dose. Surviving rings were easily distinguished as viable trophozoites at either 18-, 21-, or 24-h time points by microscopic examination of blood smears, which enabled comparisons between parasite lines. We therefore carried out concurrent collection and analysis of thin blood smears at all time points examined for flow analysis (Fig. 3A; Fig. S4). Results demonstrated that enhanced survival after the first AS dose was evident for all four P. berghei K13 mutant parasites as well as the UBP-1 mutant compared to wild-type parasites (see Fig. S5). Microscopy provided a more sensitive discrimination than flow cytometry-based estimation of clearance kinetics that was unable to distinguish mutant from wild-type parasites in the first 24 h. False positives could be due to the retention of mCherry positivity by dying parasites. For instance, we observed that a significant proportion of wild-type parasites remained mCherry positive and were counted as viable by flow cytometry (Fig. 3A; Fig. S4), whereas, microscopically, they were pyknotic forms (Fig. S5A and G). Remarkably, the M488I and R551T mutants remained smear positive after two consecutive AS doses (Fig. S5E, F, K, and L), whereas the wild-type, F458I, Y505H, and UBP-1 mutant parasites were cleared (microscopically smear negative) after 48 h. These data suggest that the M488I and R551T mutants meet the classical definition of ART resistance, as defined by the WHO based on day 3 (second generation) microscopy positivity, when accounting for the duration of the P. berghei life cycle and the dosing intervals (4). One of the four mice in the M488I treatment group remained smear positive after three consecutive AS doses (Fig. S5E). These data provide evidence that P. berghei K13 mutants modulate *in vivo* susceptibility to ARTs, resulting in a persister/delayed clearance phenotype under controlled conditions of initial parasite biomass and host immune status. Of note, we consistently used naive mice of same age, sex, breed, and genetic background.

10.1128/mBio.02312-20.4 FIG S4 Clearance kinetics of P. berghei K13 mutants upon AS treatment. Parasite clearance curves in mice with established parasitemias of K13 mutant lines following treatment with 64 mg/kg artesunate (AS) at the indicated times (see arrows) for the three cohorts. See Materials and Methods and Fig. 3A. Download FIG S4, TIF file, 2.7 MB. Copyright © 2020 Simwela et al. 2020 Simwela et al. This content is distributed under the terms of the Creative Commons Attribution 4.0 International license.

10.1128/mBio.02312-20.5 FIG S5 Microscopic analysis of P. berghei K13 mutants upon AS treatment. Microscopic analysis of Giemsa-stained thin blood smears showing preferential survival of UBP-1 (B and H) and K13 mutant parasites G1957^F458I^ (C and I), G1979^Y505H^ (D and J), G1989^M488I^ (E and K), and G2025^R551T^ (F and L) compared to wild-type parasites (A and G) upon treatment with AS. Cohorts 1 and 2 are shown in panels A to F, and cohorts 3 and 4 are shown in panels G to L. Smears were taken at time points corresponding to those shown in the clearance plots in Fig. 3A and in Fig. S4. Second and third dose treatment days are indicated by black arrows. Red arrows indicate viable parasites. Viability was deemed significant if at least 4 viable parasites were observed upon observation of at least 10 microscopic fields. Download FIG S5, TIF file, 2.8 MB. Copyright © 2020 Simwela et al. 2020 Simwela et al. This content is distributed under the terms of the Creative Commons Attribution 4.0 International license.

Another *in vivo* marker of reduced ART susceptibility in P. falciparum is the rate of recrudescence upon AS treatment, which acts as a possible indicator of AS treatment failure. However, at pharmacologically safe doses in humans (2 to 4 mg/kg), ART monotherapy treatment leads to >40% recrudescence rates (1, 3), making it difficult to use this approach to separate clinically ART-sensitive from ART-resistant parasites. P. berghei K13 mutants, therefore, provide the opportunity to test for recrudescence rates using controlled parasite inocula as well as AS or ART dose ascendency. We treated groups of mice initially infected with 10^6^ K13 mutant, ART-resistant UBP-1 mutant, or wild-type parasites with a daily ART dose of 80 mg/kg for three consecutive days. This ART dose sufficiently suppresses the P. berghei wild type at equivalent parasite inocula for up to 18 days of follow-up (46). All UBP-1 mutant infections recrudesced 11 days after the last ART dose, whereas no recrudescence (0%) was observed for the wild type (Fig. 3B; see also Table S3). These data are consistent with our previous observations (46). However, R551T mutant parasite infections achieved even faster recrudescence, namely, 50% on day 4 after the last dosing and 100% a day later, indicating a higher level of *in vivo* resistance for this K13 mutation compared to that of the UBP-1 mutant. M488I mutant parasites had a similar recrudescence profile beginning on day 6. The Y505H and F458I mutant lines both achieved recrudescence at approximately the same time as the UBP-1 mutant; however, the latter achieved only 50% recrudescence across the 18-day follow-up period (Fig. 3B; Table S3). These data further confirm that P. berghei K13 mutants modulate *in vivo* susceptibility to ARTs and, crucially, that recrudescence rates strongly correlate with our *in vitro* DHA RSA profiles (Fig. 2) as well as with *in vivo* clearance kinetics in established infections (Fig. 3A; Fig. S4 and S5).

### P. berghei K13 mutants are associated with an *in vivo* fitness cost but are preferentially selected for in the presence of AS or CQ.

**FIG 4:**
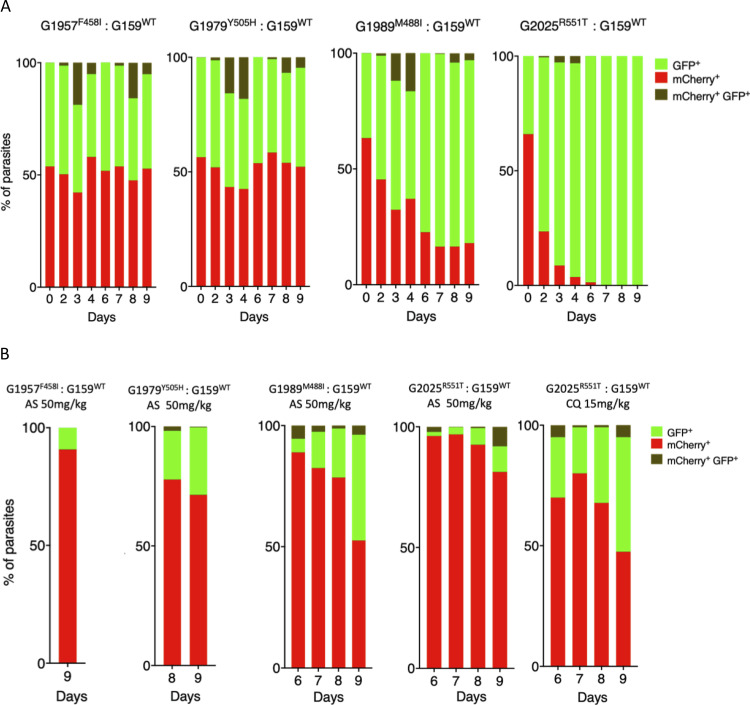
Relative fitness of P. berghei K13 mutants in presence or absence of AS or CQ. Growth competition assays with K13 mutant lines that constitutively express mCherry compared to the wild-type G159^WT^ line that constitutively expresses GFP in the presence or absence of drug pressure. The G159^WT^ line was mixed with a given mutant line at a 1:1 ratio in three groups of mice on day 0. The first group was left untreated, the second group received a dose of AS at 50 mg/kg starting from 3 h after i.p. injection for three consecutive doses, while the third group consisting of the 1804 ^WT^, G1980^V2721F^, and K13 mutant G2025^R551T^ lines received CQ at 15 mg/kg at similar dosing times as AS. Percentages of mCherry- or GFP-positive parasites were determined by flow cytometry as described in Materials and Methods. (A) Percentage population changes as measured by flow cytometry of the G1957^F458I^, G1979^Y505H^, G1989^M488I^, and G2025^R551T^ mutant lines relative to that of the G159^WT^ wild-type line. (B) Proportion representation of the G159^WT^ line in mixtures with G1957^F458I^, G1979^Y505H^, G1989^M488I^, and G2025^R551T^ lines on the days of recrudescence upon treatment with AS or CQ as indicated.

To assess the fitness of our P. berghei K13 mutants, we performed direct head-to-head competitions with wild-type parasites under *in vivo* growth conditions. P. berghei K13 or UBP-1 mutant lines or the parental 1804^WT^ (mCherry positive) line were mixed at a 1:1 ratio with the G159^WT^ (green fluorescent protein [GFP] positive) line and injected into mice. Changes in the proportion of GFP- or mCherry-positive parasites in the competition mixture were then quantified by flow cytometry over 9 days. These assays revealed that the F458I and Y505H mutant parasites were fitness neutral relative to the G159^WT^ line, whereas the M488I and R551T mutants carried significant fitness costs (Fig. 4A). Both the M488I and R551T mutations were associated with high levels of reduced susceptibility to DHA *in vitro* (Fig. 2), delayed clearance kinetics (Fig. 3A; Fig. S4), and faster recrudescence following ART treatment *in vivo* (Fig. 3B; Table S3). Comparatively, the R551T mutant parasites had a more severe growth defect than the M488I mutants and were completely outcompeted by the GFP-positive wild-type line by day 7 (Fig. 4A). This is consistent with previous observations of high *in vitro* fitness costs for the equivalent P. falciparum R539T mutation (40). In comparison to the G159^WT^ line, the parental wild-type line (1804^WT^) was fitness neutral, whereas the UBP-1 V2721F mutant carried a minor growth defect as previously observed (46) (see Fig. S6A and B). We also examined the proportions of GFP-positive versus mCherry-positive parasites over time in P. berghei K13 mutant and wild-type parasites upon treatment with AS. Mutant parasites were mixed at 1:1 ratios with the G159^WT^ line and injected into mice that were treated with AS at 50 mg/kg beginning 3 h after infection for three consecutive days. Monitoring of recrudescence up to day 9 revealed that, upon AS treatment, the M488I and R551T mixtures recrudesced slightly faster than the wild-type mixture and were highly enriched for the mutant population (>90%) at the time of recrudescence (Fig. 4B). The F458I and Y505H mutant mixtures recrudesced slightly later (Fig. 4B), as did the UBP-1 V2721F mutant (Fig. S6B), and were all significantly enriched for the mutants. In contrast, the proportions of GFP-positive versus mCherry-positive parasites in the parent 1804^WT^ and G159^WT^ competition mixture after AS treatment did not change at the time of recrudescence (Fig. S6A). These data show that mutant P. berghei K13 parasites are preferentially selected for upon AS treatment, despite some carrying growth defects that rendered them at a complete competitive disadvantage in the absence of drug.

10.1128/mBio.02312-20.6 FIG S6 Growth competition of the parent 1804^WT^ and UBP-1 V2721F mutant line compared to the G159^WT^ in the presence or absence of AS or CQ. Growth competition assays with the wild-type parent 1804^WT^ or the UBP-1 G1980^V2721F^ line compared to wild-type GFP-expressing G159^WT^ line in the presence or absence of artesunate (AS) or chloroquine (CQ) drug pressure. Parasites were mixed at a 1:1 ratio, injected into mice, and left treated or untreated with AS at 50 mg/kg or CQ at 15 mg/kg as described in Materials and Methods and Fig. 4. (A) Percentage population changes of the 1804^WT^ and wild-type G159^WT^ in the absence of drug and on the day of recrudescence on day 9 for AS or CQ. (B) Proportion representations of the G159^WT^ line in mixture with G1980^V2721F^, in the absence of drug or upon AS or CQ treatment, on the days of recrudescence. Download FIG S6, TIF file, 2.7 MB. Copyright © 2020 Simwela et al. 2020 Simwela et al. This content is distributed under the terms of the Creative Commons Attribution 4.0 International license.

With the supposed role of P. falciparum K13 in mediating parasite hemoglobin endocytosis (43–45), we also speculated that P. berghei K13 mutant parasites with strong ART resistance phenotypes might be able to modulate susceptibility to CQ (to some degree) through a similar dysregulation of the endocytic machinery. Using the *in vivo* competition assay under drug pressure as with AS as described above, the parental 1804^WT^ line, the UBP-1 V2721F line, and the K13 R551T mutant line were each mixed at 1:1 ratios with the G159^WT^ line and treated with CQ at 15 mg/kg. At the time of recrudescence, the proportion of 1804^WT^ parasites (mCherry positive) did not significantly change compared to the proportion of GFP-positive G159^WT^ parasites (Fig. S6A). In comparison, the UBP-1 V2721F mutant was enriched to ∼70% (Fig. S6B), which mirrors our previous observations that this mutation can indeed be selectively enriched by CQ (46). Interestingly, upon CQ treatment, the combination of R551T mutant parasites and the G159^WT^ line achieved recrudescence at almost the same rate as that under AS pressure, with mutant parasites enriched to ∼72% (Fig. 4B). These data suggest that K13 mutations can also contribute to low-level protection to CQ (43, 44).

### A *Plasmodium*-selective proteasome inhibitor is potent against P. berghei wild-type and K13 mutant parasites and synergizes DHA action.

**FIG 5:**
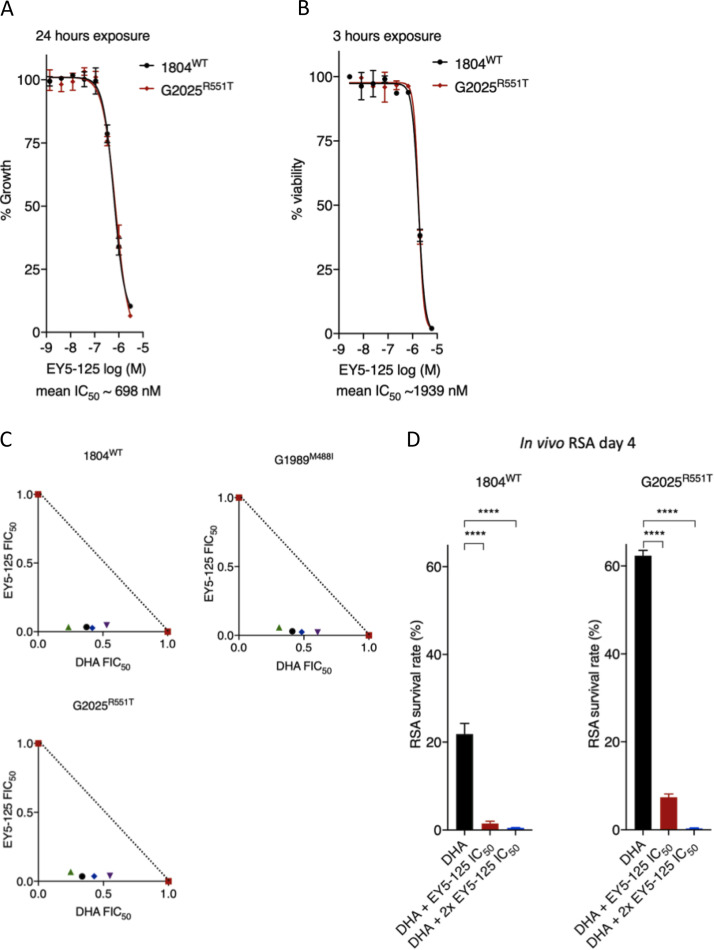
Activity and DHA synergy of proteasome inhibitor in P. berghei K13 mutants. Dose-response curves and mean IC_50_ values for the *Plasmodium-*selective proteasome inhibitor EY5-125 for the wild-type 1804^WT^ and K13 mutant G2025^R551T^ lines in standard 24-h assays (A) or 3-h exposure assays conducted on early ring-stage parasites (B). Mean IC_50_ is a calculated average for the two lines independently screened in three biological repeats. (C) Isobologram plots representing the interaction between DHA and EY5-125 in the wild-type 1804^WT^, G1989^M488I^, and G2025^R551T^ lines. Plots show mean FIC_50_ values (Table S4) for each drug calculated from three biological repeats. (D) Synergy of EY5-125 proteasome inhibitor with DHA in the *in vivo* RSA. Parasites were exposed to DMSO or DHA at 700 nM alone or in combination with EY5-125 at 3-h IC_50_ or 2× IC_50_ and then injected back into mice 24 h later as described in Materials and Methods. Parasitemias in mice infected with drug or DMSO-treated parasites were determined by flow analysis of mCherry expression on day 4 after i.v. injection and were used to calculate percent survivals relative to that of DMSO-treated parasites. Error bars are standard deviations from three biological repeats. Statistical significance was calculated using one-way ANOVA alongside the Dunnett’s multiple-comparison test. ****, *P* < 0.001.

An enhanced cell stress response characterized by upregulation of genes in the unfolded protein response (UPR) is a typical signature of ART-resistant parasites (50). Resistant parasites (K13 mutants) also display enhanced activity of the ubiquitin proteasome system (UPS), a conserved eukaryotic pathway that acts downstream of the UPR by degrading unfolded proteins (49, 55). UPS inhibitors are available for cancer treatment and have been shown to synergize DHA activity in wild-type and K13 mutant P. falciparum both *in vitro* and *in vivo*, marking them as promising agents for overcoming ART resistance (49, 56). The *Plasmodium-*selective proteasome inhibitor EY5-125 is a potent antimalarial (standard IC_50_ against P. falciparum = 19 nM) that acts in synergy with ART against both ART-resistant and -sensitive P. falciparum strains *in vitro* (57). Here, we tested the efficacy of EY5-125 against P. berghei wild-type and K13 mutant parasites and examined its potential ability to synergize DHA action. P. berghei wild-type and the most ART-resistant K13 mutant (R551T) parasites were found to be equally sensitive to EY5-125 (Fig. 5A and B). Compared to that in P. falciparum (72-h IC_50_ of ∼19 nM and 1-h IC_50_ of ∼648 nM), EY5-125 is much less potent in P. berghei in both standard *in vitro* growth inhibition (IC_50_ = ∼700 nM) and 3-h assays (IC_50_ = ∼1,900 nM), respectively (Fig. 5A and B). These differences could be due to species-specific differences in drug sensitivity as we have observed with ARTs (46, 54) and many other drugs (58). However, combinations of DHA and EY5-125 in fixed-ratio isobologram analyses revealed a strong synergistic interaction against the P. berghei K13 wild-type and M488I and R551T mutant lines (Fig. 5C; see also Table S4). We also employed our *in vivo* RSA to examine whether a combination of DHA at 700 nM and EY5-125 at the equivalent 3-h IC_50_ (1.94 μM) or 2× IC_50_ (3.88 μM) could impact parasite survival rates. Indeed, at both the 3-h IC_50_ and 2× IC_50_ concentrations, EY5-125 strongly synergized with DHA (700 nM), as evidenced by significant abrogation of survival for both the wild-type and R551T mutant lines (Fig. 5D). These data demonstrate that proteasome inhibitors synergize DHA action in P. berghei K13 mutants equally as well as wild-type parasites both *in vitro* and *in vivo* and have the potential to be used to overcome ART resistance.

## DISCUSSION

In this study, we successfully employed CRISPR/Cas9 editing to introduce four of the six targeted orthologous P. falciparum K13 (F446I, M476I, Y493H, and R539T) mutations into the *K13* gene of the rodent model of malaria P. berghei. Meanwhile, introduction of two mutations (C580Y and I543T) could not be achieved. As debate continues on the role of K13 in mediating *in vivo* susceptibility to ARTs (37), phenotyping of these P. berghei K13 (F458I, M488I, Y505H, and R551T) mutants provides experimental evidence for the ability of mutant K13 to confer *in vivo* resistance to ARTs in a naive parasite genome background. These mutants displayed reduced *in vitro* susceptibility to DHA and phenocopied P. falciparum delayed clearance phenotypes upon AS treatment. Moreover, these K13 mutants achieved faster recrudescence upon ART treatment under *in vivo* growth conditions. As in P. falciparum, certain P. berghei K13 mutations were found to cause significant growth defects, which highlights the structural and functional conservation of this protein across the two *Plasmodium* species and illustrates the fitness trade-offs that the acquisition of such mutations exerts on malaria parasite physiology.

ART resistance, principally associated with mutations in K13, is now almost endemic in Southeast Asia, with risks of global spread threatening the utility of ACTs that are at the forefront of malaria control programs (2). The P. falciparum C580Y K13 mutation is the most frequently observed (with >50% prevalence) and has reached fixation in most parts of Southeast Asia (25, 59). Why the P. falciparum C580Y mutation is so successful compared to other K13 mutations remains unclear. This mutation does not associate with high RSA survival rates compared to P. falciparum R539T or I543T mutant parasites, and treatment failure rates and parasite clearance rates are not more significant in C580Y-harboring parasites than those with other K13 mutations (27, 28, 60). Do fitness constraints, founder genetic landscapes, or species-specific differences between P. berghei and P. falciparum K13 explain our failed attempts to introduce the C592Y or I555T mutations in P. berghei? The structural homology model of the K13 propeller domain presented here demonstrates that this region is highly conserved between P. berghei and P. falciparum K13, with identical amino acids at the sites of mutations associated with ART resistance. Our unsuccessful attempts to introduce the P. berghei C592Y or P. berghei I555T mutations could therefore be more related to growth disadvantages or other deleterious effects. For example, in P. falciparum, the equivalent I543T and R539T mutations carry the most pronounced fitness costs (40), which could partly explain our inability to introduce the P. berghei I555T mutation in P. berghei. Moreover, P. berghei K13 mutations were introduced into PBANKA parasites with no history of ART exposure. These parasites might therefore be more sensitive to fitness impacts conferred by the P. berghei I555T or P. berghei C592Y substitution, as introduction of the equivalent P. falciparum C580Y in parasites isolated before ART was clinically introduced carried significant growth defects, as opposed to more recent Cambodian isolates where it was fitness neutral (40). A less prevalent *K13* allele, P. falciparum R561H, that associates with significant delays in parasite clearance and peaked in prevalence in 2012 in Southeast Asia but has since declined (60) also easily outcompeted the P. falciparum C580Y mutation in head-to-head competitions (42). These data suggest that acquisition and propagation of certain P. falciparum *K13* alleles, notably the C580Y substitution, might require appropriate founder genome architectures to compensate for the deleterious phenotypes. In these situations, K13 mutations (P. falciparum C580Y, for example), would arise in a necessary compensatory background that mitigates the deleterious growth effects leading to an initial soft sweep. In the case of ACTs, these compensatory backgrounds may also serve as general templates upon which partner drug resistance mutations might arise. This seems to be the case with the recent aggressive expansion of parasite colineages carrying the P. falciparum C580Y mutation and piperaquine resistance determinants (10, 11).

Despite the obstacles to introducing the P. berghei C592Y and I555T mutations, introduction of the P. falciparum R539T equivalent was achieved in P. berghei (R551T) despite low editing efficiency in the initial transfection. We were, however, able to enrich for this mutation with AS selection applied *in vivo*, yielding almost clonal levels of the P. berghei R551T mutant. Similar to the P. falciparum R539T mutant, clonal P. berghei R551T mutant parasites carried the strongest DHA resistance phenotypes *in vitro* as well as the clearest AS or ART resistance profiles *in vivo*. The P. falciparum R539T and P. falciparum I543T mutations occur at relatively low frequencies in Southeast Asia, with the prevalence of both mutations ranging between 0.3% and 3.5% (36, 41, 59). This could be due to the pronounced fitness cost of these mutations (40) limiting their expansion, which we also observed with the P. berghei R551T mutant parasites. The combination of a naive genomic background and species-specific differences can also be invoked to explain some phenotypic differences (growth rate and level of ART resistance) seen between mutant lines of P. falciparum and P. berghei K13, as observed in this study. For example, P. falciparum Y493H mutants clearly associate with increased RSA survival (23, 28) and delayed parasite clearance phenotypes (23, 41, 61), unlike the P. berghei counterpart (Y505H) that displayed low-level resistance to ARTs *in vitro* (in the standard assay but not in the adapted RSA) and *in vivo*. This could be due to additional underlying genetic factors in P. falciparum isolates providing an additive effect to the observed phenotypes, which would be absent in P. berghei. Nevertheless, the other P. berghei K13 mutations tested here appear to directly reflect the impact of the equivalent mutations in P. falciparum. Both P. berghei F458I (this study) and P. falciparum F446I K13 mutants are fitness neutral (62) and do not enhance RSA survival *in vitro* (62, 63) yet carry ART-protective phenotypes *in vivo* (64–66). Furthermore, P. berghei M488I K13 mutants display a significant growth defect that has not yet been characterized in the P. falciparum equivalent (M476I) and might explain its relative scarcity in Southeast Asia (67, 68).

Enhanced proteostasis is a characteristic signature of P. falciparum K13 ART-resistant parasites, which is typified by upregulation of genes in the UPR as well as enhanced activity of the UPS (49, 50, 55). Inhibition of the UPS by 26S proteasome inhibitors synergizes DHA action both *in vitro* and *in vivo*, which has offered a potential avenue to overcome ART resistance (49). Despite UPS inhibitors (which are clinically available for treatment of certain cancers) displaying activity in malaria parasites and synergizing DHA action, their translation into animal studies has been limited by host toxicity (69, 70). Recent structure-based design of *Plasmodium-*selective proteasome inhibitors has yielded vinyl sulfone inhibitors with a wider therapeutic window and improved host toxicity profiles (56, 57). These inhibitors not only display activity in diverse P. falciparum backgrounds, including those harboring K13 mutations, but also strongly synergize with DHA (71). Even though P. berghei proteasome structures have not been solved, functional and life cycle conservation between this parasite and P. falciparum is pronounced. Using EY5-125, an inhibitor selective for the P. falciparum proteasome (57), we demonstrate similar activity and synergy with DHA in P. berghei wild-type and K13 ART-resistant mutants. Importantly, we demonstrate these properties *in vivo*, which significantly strengthens the potential of these compounds in overcoming ART resistance in infected hosts.

In conclusion, our work provides robust experimental evidence that K13 mutations modulate *in vitro* and *in vivo* susceptibility to ARTs in the P. berghei rodent model of malaria. The cause and effect link between P. falciparum K13 mutations and reduced ART susceptibility is strong (23, 28). However, the reason for ART clinical failure has remained obscure because, in some cases, delayed parasite clearance phenotypes have been reported in parasites carrying wild-type *K13* alleles (35, 72). This lack of clarity is further compounded by a reduced correlation between K13 mutations and parasite clearance half-lives or the frequencies of recrudescence in certain cases of ART monotherapy (35). As we demonstrate in this study, some of these observations may be attributable to fitness defects in mutant parasites that could confound the interpretation of recrudescence rates. These fitness differences might be especially relevant at the relatively low ART doses used in humans, which are already known to permit higher rates of recrudescence (3). Although a recent genetic cross between a P. falciparum K13 C580Y mutant parasite and an *Aotus*-infecting K13 wild-type parasite demonstrated a lack of association of this mutation with *in vivo* ART resistance metrics (recrudescence and clearance half-life) (37), we propose that this could be due to (i) the AS doses used being insufficiently high to clearly separate the lineages, (ii) the small sample sizes used, and (iii) the inherent limitation of using heterogeneous *Aotus* monkeys with various individual histories of parasite exposure and spleen status (spleen intact or splenectomized). Our *in vitro* and *in vivo* phenotypes for the P. falciparum F446I, M476I, Y493H, and R539T K13 mutation equivalents in P. berghei support their direct involvement in mediating resistance to ARTs. Our data also provide a robust immune-replete rodent host model to test for synergistic antimalarial combinations that can restore ART efficacy and overcome resistance.

## MATERIALS AND METHODS

### CRISPR/Cas9 generation of P. berghei K13 mutant lines.

The Cas9 plasmid ABR099 was used to target mutations of interest into the P. berghei *K13* locus (PlasmoDB gene identifier [ID] PBANKA_1356700) (46). To obtain P. berghei equivalents of P. falciparum ART-resistant K13 mutations (PlasmoDB gene ID PF3D7_1343700), the amino acid sequences of the two proteins were retrieved and aligned using Clustal Omega (73). To structurally align the equivalent mutations in P. berghei K13, three-dimensional homology models of P. berghei and P. falciparum K13 were constructed using SWISS-MODEL (PDB template 4zgc.1.A) for amino acid residues 362 to 738 for P. berghei and 350 to 726 for P. falciparum. Models were visualized using pyMol 2.3. sgRNAs designed to target a region within 0 to 30 bp of the mutation of interest within the P. berghei *K13* locus were initially cloned into the ABR099 plasmid (see Fig. S2A in the supplemental material). Donor DNA repair templates were designed to carry the mutation of interest in addition to silent mutations that introduced restriction sites for RFLP and that inactivated the PAMs. These templates were generated by overlap extension PCR (74) and were subsequently cloned into ABR099 plasmids carrying corresponding sgRNAs at the linker sites (Fig. S2A). Generated plasmids and all corresponding sgRNAs are listed in Table S1 in the supplemental material.

### Parasite lines and animal infections.

This study employed two P. berghei ANKA-derived parasite lines, 1804cl1 and G159. The 1804cl1 (75) and G159 (Katie Hughes, unpublished) lines express mCherry and GFP, respectively, under the control of the strong constitutive *hsp70* promoter. Infections were carried out in female Theiler’s Original mice (Envigo), 6 to 8 weeks old, weighing 25 to 30 g. Infections were established either by intraperitoneal (i.p.) injections of ∼200 μl of cryopreserved parasite stocks or by intravenous (i.v.) injections of purified schizonts or mixed-stage parasites diluted in phosphate-buffered saline (PBS). Parasitemias in infected mice were monitored by microscopic examination of methanol-fixed thin blood smears stained with Giemsa (Sigma) or flow cytometry-based analysis of infected blood stained with Hoechst 33342 (Invitrogen). Blood from infected mice was collected by cardiac puncture under terminal anesthesia. All animal work was performed in compliance with UK home office licensing (project reference P6CA91811) and ethical approval from the University of Glasgow animal welfare and ethical review body.

### Transfections.

Primary transfections were carried out in the 1804cl1 line. Approximately 10 μg of episomal plasmid DNA from the vectors described above (Table S1) was transfected by electroporation of Nycodenz-purified schizonts using the Amaxa Nucleofector Device II program U-o33, as previously described (76). Parasites were then immediately i.v. injected into mice. Positive selection of transfected parasites was commenced 24 h later by adding pyrimethamine (0.07 mg/ml; Sigma) to their drinking water.

### Genotyping of transformed parasites.

Parasite pellets were prepared from infected mouse blood that was lysed by resuspension in 1× E-lysis buffer (Thermo). Genomic DNA was extracted from the pellets using the Qiagen DNeasy blood and tissue kit according to the manufacturer’s instructions. Initial analysis of the transfected or cloned parasite lines was performed using a dual PCR-RFLP approach. PCR using primers exterior to the donor templates (Table S1 and S2) was used to amplify fragments from the genomic DNA of the mutant lines, followed by restriction digests with the artificially introduced RFLP restriction enzymes. Relative transformation efficiencies were estimated by densitometric quantification of wild-type and mutant RFLP fragments by ImageJ2 (77). Mutations and initial RFLP analyses were further confirmed by Sanger DNA sequencing.

10.1128/mBio.02312-20.8 TABLE S2 List of primers used in the study and their descriptions. Download Table S2, XLSX file, 0.1 MB. Copyright © 2020 Simwela et al. 2020 Simwela et al. This content is distributed under the terms of the Creative Commons Attribution 4.0 International license.

10.1128/mBio.02312-20.9 TABLE S3 Recrudescence of P. berghei K13 and UBP-1 mutants compared to that of the wild type in infected mice treated with ART at 80 mg/kg as described for Fig. 3B. Groups of three or four mice (M0 to M4) were infected with ∼10^6^ parasites on day 0 and treated from 3 h with ART at 80 mg/kg as indicated by arrows. A recrudescent event was recorded as “−” for negative smears or “+” with associated parasitemia (% infected erythrocytes). Refers to Fig. 3B. Download Table S3, XLSX file, 0.1 MB. Copyright © 2020 Simwela et al. 2020 Simwela et al. This content is distributed under the terms of the Creative Commons Attribution 4.0 International license.

10.1128/mBio.02312-20.10 TABLE S4 ΣFIC_50_ values for DHA and EY5-125 drug combination ratios in the 1804^WT^, G1989^M488I^, and G2025^R551T^ lines. ΣFIC_50_ values are derived from FIC_50_ data as plotted in Fig. 5C. Download Table S4, XLSX file, 0.1 MB. Copyright © 2020 Simwela et al. 2020 Simwela et al. This content is distributed under the terms of the Creative Commons Attribution 4.0 International license.

### Antimalarial agents.

DHA (Selleckchem) at 10 mM was diluted to a working concentration in schizont culture medium. The *Plasmodium-*selective proteasome inhibitor EY5-125, also known as compound 28 (57), was used to test for proteasome inhibitor synergy with DHA in K13 mutant and wild-type parasites. For *in vivo* drug treatment, AS (Sigma) was dissolved in 5% sodium bicarbonate prepared in 0.9% sodium chloride. CQ diphosphate (Sigma) was dissolved in 1× PBS. ART (Sigma) was prepared at 50 mg/ml in a 1:1 mixture of DMSO and Tween 80 (Sigma) and diluted 10-fold in sterile distilled water immediately before administration. All drugs were prepared fresh before *in vivo* administration, and drug delivery was carried out by i.p. injection.

### Twenty-four-hour P. berghei *in vitro* culture and drug susceptibility assays.

*In vitro* culture and drug susceptibility assays were carried out beginning with synchronized ring-stage parasites over 24-h schizont maturation cycles, as P. berghei can only be maintained for one intraerythrocytic developmental cycle *in vitro*. Parasites were cultured and exposed to drugs as previously described (46), after which schizont maturation was analyzed by flow cytometry. Infected cells were stained with the DNA dye Hoechst 33258. Schizont maturation was used as a surrogate marker of growth inhibition and was quantified based on Hoechst 33258 fluorescence intensity or mCherry expression. To determine growth inhibition and calculate half-maximal inhibitory concentrations (IC_50_s), the percentage of schizonts in no-drug controls was set to 100% growth, and subsequent growth percentages in the presence of drugs were calculated accordingly. Dose-response curves were plotted in GraphPad Prism.

### Adapted P. berghei ring-stage survival assays.

The P. falciparum RSA was adapted for P. berghei to further assess the *in vitro* phenotypes of K13-mutant parasites based on a previously published protocol (22). Schizonts were obtained from *in vitro* cultured parasites as previously described (76) and injected i.v. into naive mice to obtain synchronous *in vivo* infections containing >90% rings at parasitemias of 0.5% to 1.5%. Approximately 1.5 h postinjection, blood was collected from the infected mice, adjusted to 0.5% hematocrit, and exposed to 700 nM DHA or 0.1% DMSO (Thermo Fisher Scientific) in 96-well plates or 10-ml culture flasks. The plates and flasks were incubated with drug under standard culture conditions for 3 h, after which, the drug was washed off at least three times. Parasites were then returned to standard culture conditions in new plates and flasks with fresh schizont medium for *in vitro* maturation. After 24 h of incubation, parasite survival was assessed by flow cytometry analysis of Hoechst 33258-stained infected cells. Viability was assessed by gating on the Hoechst 33258 DNA stain and live mCherry expression. DHA-treated samples were compared to DMSO-treated controls processed in parallel. Percent survival was calculated using the following formula: survival (%) = (viability [%] [DHA − treated])/(viability [%] [mock DMSO − treated]).

To improve the robustness of the viability readouts beyond the 24-h flow cytometry counts, an *in vivo* expansion of the 3 h DHA- or DMSO-exposed parasites was used for selected mutants and the wild-type control. After 24 h of recovery, 2 ml of DHA- or DMSO-treated parasites was pelleted and resuspended in a 1-ml volume, from which, 200 μl was injected i.v. into mice. *In vivo* parasitemias were quantified on day 4 postinjection, from which percentage survivals based on *in vivo* parasitemia (absolute counts of mCherry positive parasites) were calculated using the following slightly modified formula: survival (%) = (parasitemia [DHA − treated])/(parasitemia [mock DMSO − treated]).

### *In vitro* isobologram drug combinations.

DHA and EY5-125 drug interaction analyses in fixed ratios were carried out using a modified fixed-ratio interaction assay as previously described (78). DHA and EY5-125 combinations were prepared in molar concentration combination ratios of 5:0, 4:1, 3:2, 2:3, 1:4, and 0:5 and were dispensed into 96-well plates. This was followed by a 3-fold serial dilution with precalculated estimates to ensure that the test wells containing the 3-h IC_50_s of the two drugs were located near the middle of the plate. The drug combinations were then incubated with synchronized ∼1.5-h-old ring-stage wild-type or K13 mutant parasites for 3 h, after which, the drugs were washed off at least 3 times. Percent viability was quantified 24 h later by flow cytometry analysis of Hoechst 33258-stained infected cells and mCherry expression. Dose-response curves were calculated for each drug alone or in combination, from which fractional inhibitory concentrations (FIC_50_) were obtained and summed to obtain the ∑FIC_50_ using the following formula: ΣFIC_50_ = (IC_50_ of DHA in combination/IC_50_ of drug DHA alone) + (IC_50_ of EY5-125 in combination/IC_50_ of EY5-125 alone).

An ΣFIC_50_ of >1 was used to denote antagonism, ΣFIC_50_ <1 synergism, and ΣFIC_50_ = 1 additivity. FIC_50_ values for the drug combinations were plotted to obtain isobolograms for the drug combination ratios.

### *In vivo* drug assays.

**(i) Parasite clearance.** Parasite clearance upon treatment with AS was used to evaluate potential delayed clearance phenotypes in K13 mutant parasites. These studies were based on a modified Rane’s curative test in established mice infections as previously described (79). Donor mice were infected with mutant lines and the wild-type control. Once a parasitemia of ∼2% was reached, blood was obtained from the donor mice and diluted in 1× PBS. Approximately 10^5^ parasites were inoculated in 4 cohorts of mice (4 mice per line) by i.p. injections on day 0, and parasitemias were allowed to rise to ∼10%, typically on day 5. On day 5, at time zero, 2 μl of blood was collected and diluted 200-fold in 1× PBS. Thin blood smears were also collected at this time. All four cohorts were then dosed with AS at 64 mg/kg at 0, 24, and 48 h. Blood sampling was performed for flow cytometry analysis, and thin blood smears were prepared five times during the first 24 h for each cohort and at least daily thereafter in a staggered manner that allowed for a 3 h life cycle coverage in the first 24 h for at least two cohorts. Parasite density at each time point was determined by absolute cell counts and mCherry expression in 0.1 μl of whole blood diluted in PBS analyzed on a MACSQuant Analyzer 10. Thin blood smears of parasite morphologies were analyzed by microscopy. Significant viability counts in microscopy smears were based on microscopic confirmation of at least four viable parasites in a minimum of 10 fields. Clearance kinetics of normalized parasite densities versus time were plotted in GraphPad prism.

**(ii) Recrudescence.** A modified Peters’ 4-day suppressive test was used to assess *in vivo* response profiles and recrudescence rates of wild-type and mutant lines as previously described (46, 80). Infections were initiated by i.p. inoculation of 10^6^ parasites diluted from donor mice and were followed by three daily consecutive drug doses of ART at 80 mg/kg, with the first initiated ∼3 h postinoculation. Parasitemia was monitored by microscopic analysis of methanol-fixed Giemsa-stained smears up to day 18 or until recrudescence was observed.

### *In vivo* growth competition assays in presence or absence of drug treatment.

Mutant lines in the 1804cl1 mCherry background line were mixed with the G159 GFP line at 1:1 ratios and injected i.p. (total parasite inocula of 10^6^) into 3 groups of mice. The groups were either left untreated or treated with AS at 50 mg/kg for 3 consecutive days starting 3 h postinfection or CQ at 15 mg/kg. Parasitemias and fractions of mutant versus wild-type parasites were determined by flow cytometry-based quantification of mCherry- or GFP-positive parasite populations.

### Reagent availability.

Parasite lines and plasmids are available upon request from A. Waters.
