# Supplementary figures and images for: Plasmodium berghei K13 Mutations Mediate In Vivo Artemisinin Resistance That Is Reversed by Proteasome Inhibition
Source: mBio. 2020 Nov 10;11(6):e02312-20. doi: 10.1128/mBio.02312-20 (PMC7667033; doi:10.1128/mBio.02312-20)

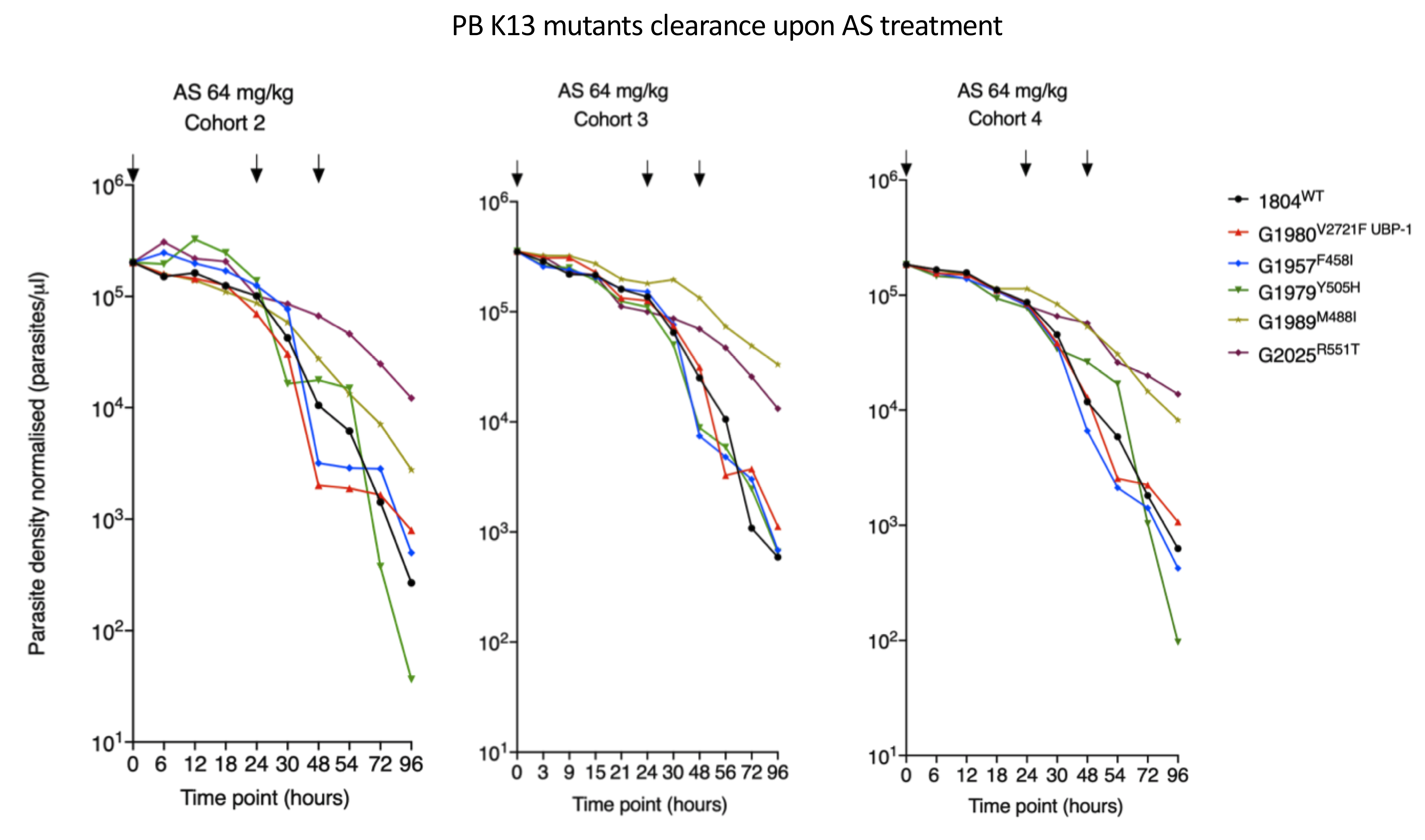

Supplement: FIG S4 [file mBio.02312-20-sf004.tif]
